# The Connection of Burns with Ulceration of the Duodenum

**Published:** 1890-04-12

**Authors:** 


### SURGERY.

### THE CONNECTION OF BURNS WITH ULCERATION OF THE DUODENUM.

Mr. Southam has given a case of this kind in the *Lancet.* A man was severely burnt about the face, arms, and thighs, the injuries proving fatal on the twelfth day. For the first eleven days the temperature recorded was 103·2 deg., rising in the last twenty-four hours of life to 110·2 deg. During the last few days he complained of pain and tenderness in the epigastric region. Otherwise there were no special features in connection with the case, nor were there any other symptoms indicative of duodenitis. At the necropsy, two well-defined ulcers were found close to the pylorus. Dr. Hunter has theorized that the duodenitis is due to the excretion through the bile of some irritant matter formed from the disintegration of the burnt tissues, which is perhaps only generated in certain cases, and the presence of which in the system is attended by marked pyrexia. Now, although every surgical author mentions ulcer of the duodenum as a sequence of burns, we do not recollect having seen any attempted explanation previously. Ulceration is generally manifested when it does occur between the 10th and 14th day by vomiting or purging of blood. It is, however, worthy of note that ulceration of the lower part of the œsophagus has also been observed in connection with burns. And still further, death may result from gastritis, ulcer of the stomach, peritonitis, pneumonia, pleurisy, bronchitis. There is, indeed a great tendency to hyperæmia and inflammation of important organs. The real reason of this is, perhaps, to be found in the skin destruction caused by the burn, functions which the skin should perform being thrown on other organs. It would appear that under such circumstances any organ being constitutionally or otherwise predisposed to disease would most probably suffer. After burns death from ulceration of the duodenum is not more common than death from congestion or inflammation of other internal organs. But the matter is one which requires further investigation.

